# Supplementary material for: Spatial–Temporal Evolution and Improvement Measures of Embodied Carbon Emissions in Interprovincial Trade for Coal Energy Supply Bases: Case Study of Anhui, China
Source: Int J Environ Res Public Health. 2022 Dec 18;19(24):17033. doi: 10.3390/ijerph192417033 (PMC9779169; doi:10.3390/ijerph192417033)
Supplement: Supplementary file 1 [file ijerph-19-17033-s001.zip › ijerph-2073370-supplementary.pdf]

Table S1 Comparison table of major abbreviations that appears in the main text.

Full name	Abbreviation
carbon emissions	CEs
embodied carbon emissions in interprovincial trade	ECEs-IPT
commodity value	CV
economic integration regions	EIRs
energy supply bases	ESBs
regional economic integration	REI
Yangtze River Economic Belt	YREB
Yangtze River Delta	YRD
input-output	IO
multi-regional input-output	MRIO

Table S2 Comparison table of 28 industry sectors code, full name and abbreviation.

Industry sectors code	Industry sectors full name	Abbreviation
S01	farming, forestry, animal husbandry, and fisheries	Agri
S02	coal mining and washing	Coalmin
S03	petroleum and natural gas extraction	CrudeOil
S04	metal ores mining and dressing	MetalOreMin
S05	nonmetal mineral ores mining and dressing	NonMetalOreMin
S06	food manufacturing and tobacco processing	FoodTobacco
S07	textile industry	Textile
S08	apparel, leather, and related products	Apparel
S09	wood processing and furniture manufacturing	WoodFurniture
S10	papermaking, printing and paper product manufacturing	PaperCulture
S11	petroleum processing, coking, and nuclear fuel processing	RefPetrol
S12	chemicals and medicinal products	Chemical
S13	nonmetal mineral products	NonMProd
S14	metal smelting and rolling processing	MetalSmelt
S15	metal products	MetalProd
S16	ordinary and special machinery manufacturing	Machinery
S17	transportation equipment manufacturing	TranspEq
S18	electric equipment and machinery manufacturing	ElecMachinery
S19	electronic and telecommunications equipment manufacturing	ElectronicEq
S20	instrumentation and cultural office machinery manufacturing	MeasureInstr
S21	other manufacturing industry	OtheManuf
S22	electricity and heat production and supply	ElectpowerProd
S23	gas production and supply	Gas
S24	water production and supply	Water
S25	construction	Construct

S26	transportation, storage, and post and telecommunication services	Transport
S27	wholesale and retail trade, catering services	WholesRetail
S28	other services	Other

Note: Industry names in the main text appear in abbreviations.
